# Expression of MUS81 Mediates the Sensitivity of Castration-Resistant Prostate Cancer to Olaparib

**DOI:** 10.1155/2022/4065580

**Published:** 2022-07-21

**Authors:** Lifeng Gong, Yu Tang, Li Jiang, Wei Tang, Shengjun Luo

**Affiliations:** Department of Urology, The First Affiliated Hospital of Chongqing Medical University, Chongqing 400010, China

## Abstract

This project attempts to clarify the expression of MUS81 in castration-resistant prostate cancer (CRPC) and the effect on drug sensitivity to Olaparib. We collected clinical surgical samples of patients who were suffering from benign prostatic hyperplasia (BPH), common prostate cancer (PCa), and castration-resistant prostate cancer (CRPC) and detected the expression of MUS81 in healthy prostate epithelial cells, PCa cells, and androgen-independent PCa cells. We subsequently performed CCK-8 assays, flow cytometry, and Transwell invasion and migration assay to determine the proliferation, apoptosis, invasion, and metastasis abilities of transfected CRPC cells as well as drug toxicity of Olaparib to CRPC cells. The expression of MUS81 indicated marked upregulation in PCa and CRPC tissues, compared with the level of MUS81 in BPH tissues. MUS81 silencing inhibited the proliferation of CRPC cells and promoted their sensitivity to Olaparib. MUS81 silencing in CRPC cells remarkably accelerated cell apoptosis and greatly inhibited cell invasion and metastasis after Olaparib administration. MUS81 silencing in CRPC cells has significantly enhanced the sensitivity of cells to Olaparib, which provides evidence for the prediction of Olaparib resistance in CRPC cells by the MUS81 gene and is expected to become a promising gene target in CRPC therapy.

## 1. Introduction

As a frequent tumor affecting males in many developed countries [1, 2], PCa has been reported with increasing cases in China as well in recent years [3, 4]. Despite the survival time of patients that may exceed 15 years in early diagnosis and confirmation [5], the majority of them seek medical treatment in advanced stages leading to a shorter survival time [3]. At present, androgen deprivation therapy has been a principal option for PCa, whereas advanced PCa patients inevitably develop into CRPC [6]. As for CRPC, the exploration of novel therapeutic targets of genes and protocols has emerged as a research hotspot due to the absence of effective treatment.

MUS81 was discovered by scholars Interthal and Heyer in 2000 [7]. The gene encodes a structure-specific endonuclease, which solves problems of Holliday Junctions and it is essential for repairing DNA double-strand break and maintaining chromosome integrity by constructing a heterodimer with Eme1/Mms4 [8–11]. McPherson et al. [12] attested that the death of 73% MUS81^−/−^ mice and 50% of MUS81^+/-^ mice was due to diverse spontaneous tumors, e.g., breast cancer, lymphoma, and PCa, suggesting that MUS81 may function as an effective tumor suppressor in mice. Some researchers have found that the expression of MUS81 is downregulated in human hepatocellular carcinoma, gastric cancer, and colorectal cancer [13–15], and its SNP links to increased risks of breast cancer [16], but its expression was upregulated in ovarian cancer tissues [17–19]. Despite such research indicating differential expression of MUS81 in different human tissues and potential roles in malignant tumors, its expressions and roles in CRPC remain to be determined. Evidence has proven that tumor suppressor gene p53 acts as a key observation site of MUS81 participating in the DNA repair pathway [20], and checkpoint genes including ATM [21], Cds1/chk2, and Rhp51/Rad51 have also been involved in the DNA repair pathway of MUS81 [20, 22, 23].

Olaparib functions as a poly ADP-ribose polymerase inhibitor (PARPi). The poly ADP-ribose polymerase (PARP) is an enzyme involved in excising and repairing DNA single-strand break bases, while PARPi inhibits the activity and elicits disturbance of DNA repair and accumulation of damage, thereby facilitating apoptosis of tumor cells [24]. Phase III clinical trials have indicated that Olaparib produced a significant response in CRPC patients with prolonged progression-free survival time [25]. A recent study has exhibited that the DNA damage repair pathway participates in the nonandrogen receptor therapy of CRPC [6]. The MUS81 gene also links to DNA repair genes, namely, ATM and Cds1 as mentioned previously. Accordingly, it is speculated that MUS81 may affect the growth, invasion, metastasis, and apoptosis of CRPC cells by mediating DNA repair.

The objective of this research is to elucidate if the expression of MUS81 affects the sensitivity of CRPC to Olaparib by identifying the expression of MUS81 in clinical specimens of CRPC patients and conducting a series of cell experiments in vitro after lentivirus transfection, hoping to provide novel gene targets and therapeutic insights in treating CRPC.

## 2. Materials and Methods

### 2.1. Collection of Clinical Surgical and Pathological Tissues

Pathological specimens were obtained from patients who accepted either electroprostatectomy or radical prostatectomy in the Department of Urological Surgery of the First Affiliated Hospital of Chongqing Medical University from 2013 to 2018. Among them, there were 40 cases of BPH, 45 cases of PCa, and 15 of CRPC. Pathological tissues were embedded in paraffin with 4% paraformaldehyde initially, and then, immunohistochemical staining was performed. Procedures of the above surgeries were conducted in line with the 2005 AUA Operation Guidelines. The collection of relevant pathological tissues was given approval by the Ethics Committee of the First Affiliated Hospital of Chongqing Medical University.

### 2.2. Blind Semiquantitative Analysis of Immunohistochemical Staining

The antigen in tissues was determined by a mixture of the biotin-labeled secondary antibody, streptomycin antibiotic protein-linked peroxidase, and stromatic, that is, immunohistochemical streptavidin peroxidase (SP) method. The primary antibody (MUS81 antibody, 1 : 100, BM6818, IGEE, China) was incubated overnight at 4°C. Chromogenic working solution (freshly prepared for use) was prepared for DAB coloration. After the coloration, an observation was made under a light microscope. Then, final experimental results were photographed and recorded. In a semiquantitative analysis, the expression of the MUS81 protein in immunohistochemical staining sections was scored by two pathologists, and the results were all taken as the average. The semiquantitative scoring rules were as follows: (1) Percentage of MUS81 expression-positive cells under the microscope was categorised as 0% = score 0, <25% = score 1, 25–49% = score 2, 50–74% = score 3, and 75–100% = score 4. (2) Staining intensity of the MUS81 protein was divided into nonstaining, weak staining, general staining, and strong staining, and the corresponding score was 0, 1, 2, and 3 points. The scoring formula was as follows: total scores of immunohistochemical staining = percentage of positive cells × staining intensity (scores multiplied). Meanings of the total staining scores presented as follows: 0 scores represented negative (-), 1-3 scores represented weak positive (+), 4-6 scores represented positive (++), and 8-12 scores represented strong positive (+++). To generate objective and realistic results, we randomly selected 10 visual fields from each section under microscopic observation and scored by pathologists through discussions, and then, an average total score was applied [26].

### 2.3. Cells

Normal human prostate epithelial cells RWPE-1, prostate cancer cells LNCap, and nonandrogen-dependent prostate cancer cells PC3 and DU145 were provided by the Shanghai Cell Bank of the Chinese Academy of Sciences.

### 2.4. Cell Transfection and Screening of Stable Transformants

The following were grouped: blank control group (PC3/DU145), MUS81 overexpression group (LV), negative virus control group (BV), MUS81 interference group (sh), and virus no-load group (EV). By reviewing relevant literature and preliminary experimental results, we obtained the minimum effective screening concentration and the optimal MOI value of puromycin. The summary of the experimental experience revealed that the higher the cell density of lentivirus transfection, the higher the transfection efficiency and expression efficiency. When the cell culture density was ranged in 60%-80%, the culture medium was replaced containing lentivirus, Lipofectamine™ 2000, and 5 *μ*g/mL polybrene corresponding to cell density. Transfection cells were screened with 2 *μ*g/mL puromycin after 72 h. After continuous culture for 72 h, the selected cells were observed under an optical fluorescence microscope, and successfully transfected cells displayed green fluorescence under the microscope. The transfection rate of cells in each group was observed and recorded. The successfully transfected cells were cultured and passaged, and stable transformants were obtained [19].

### 2.5. Western Blot

The transfected cells were cultured and collected from each group; meanwhile, the protein was extracted as well. Protein concentration and quantification were subjected to BCA assay. After determining the amount of loading sample protein, SDS polyacrylamide gel electrophoresis and membrane transfer were performed. The PVDF film was sealed with rapid sealing liquid after electroporation. Primary antibodies were supplemented and cultured in a shaker at 4°C overnight. Subsequently, following 1 h incubation by supplementing of secondary antibodies at room temperature for 1 h, ECL development was carried out to obtain target strips and internal reference strip images. The antibody information is as follows: MUS81, 1 : 500 (BM6818, IGEE, China); ATM, 1 : 500 (BM19650, IGEE, China); p-ATM, 1 : 500 (BMP1030, IGEE, China); caspase-3, 1 : 500 (BM62156, IGEE, China); Bcl-2, 1 : 1000 (BM19693, IGEE, China); BAX, 1 : 1000 (BM19684, IGEE, China); and GAPDH, 1 : 5000 (BM19056, IGEE, China).

### 2.6. qRT-PCR Assay

The design and synthesis of PCR primers used in this experiment were completed by the Takara Company of the United States. MUS81 included the following: upstream primers 5′-AGAGGTGTGCTCAGAAGTCC-3′ and downstream primers 5′-TTCTGACTCGGCCAACTTCT-3′. The length of the amplified product was 184 bp. Internal reference *β*-actin included the following: upstream primers 5′-CATGTACGTTGCTATCCAGGC-3′ and downstream primers 5′-CTCCTTAATGTCACGCACGAT-3′. The length of the amplified product was 250 bp. Total RNA was extracted with a TRIzol reagent (Invitrogen, Carlsbad, CA). RNAs were converted to cDNAs using a reverse transcription kit (Takara, Japan) and SYBR® SYBR Green PCR kit (Takara, China) to measure mRNA expressions, and the mRNAs were quantified through the -*ΔΔ*Ct method. When RNA in the tube was transparent and visible, it was diluted with 20 *μ*L of DEPC water completely. The concentration of extracted RNA was identified by an ultraviolet spectrophotometer, which was also used to determine the concentration of cDNA after reverse transcription. PCR experimental conditions were set for predenaturing at 94°C for 2 min, annealing extension at 60°C for 30 s, and 30 s at 72°C with 35 cycles. The final extension was performed at 72°C for 2 min. In this experiment, three multiple pores were set for each sample. The average absorbance value was taken as the final value. The relative quantitative method was adopted to calculate the altered mRNA expression of MUS81.

### 2.7. Detection of the Proliferation Ability and Drug Toxicity of Cells by CCK-8 Assay

Olaparib solution (50 mM) preparation was as follows: 10 mg Olaparib and DMSO 0.4603 mL were dissolved fully and stored at 4°C. Detection of cell proliferation was as follows: each well of 96-well plates was supplied with 100 *μ*L medium containing 500 cells and 4 multiple pores in each group. After being cultured for 24 h, 48 h, and 72 h, the cells of each well were supplemented with10 *μ*L of CCK-8 cell proliferation detection reagent and followed by incubation at 37°C for 1-4 h. The optical density (OD) of each well was detected at 450 nm using a microplate reader. Taking the *x*-axis as the cell culture time and the *y*-axis as the OD value, a cell proliferation curve was plotted based on the measured data. Detection of the drug toxicity of Olaparib to cells was as follows: the method of laying board was identical, and four different concentrations of Olaparib were set for each group of cells. An equal volume of Olaparib dissolved in dimethyl maple (DMSO) was supplied to the control group to the experimental group, cultured in an incubator containing 5% CO_2_ at 37°C for 48 h. After the addition of the 10 *μ*L CCK-8 drug toxicity detection reagent, incubation was performed at 37°C for 1-4 h. The OD value was detected at 450 nm using the reader. The formula applied for calculating the cell survival rate was as follows: cell survival rate (%) = [*A*(treated with drug) − *A*(blank)]/[*A*(untreated with drug) − *A*(blank)] × 100%, where *A*(treated with drug) represented the OD value of well-containing cells, CCK-8 detection reagent, and Olaparib or DMSO solutions; *A*(untreated with drug) was the OD value of well-containing cells and CCK-8 detection reagent, but without Olaparib or DMSO solutions; and *A*(blank) was the OD value of a well containing CCK-8 detection reagent and culture medium but without cells. The cytotoxicity curve was plotted in line with the results as the *x*-axis for drug concentration and the *y*-axis for the cell survival rate. The above experiments were repeated 3 times or above.

### 2.8. Detection of Cell Apoptosis by Flow Cytometry

The experiment was divided into four groups: the sh group represented MUS81 knockout cells, the EV group represented the no-load virus group, the sh+Olaparib group, and the EV+Olaparib group represented MUS81 knockout cells, and no-load virus cells were treated with 80 *μ*L Olaparib for 48 h. The supernatant of each group was collected, digested with a quantity of trypsin, and then centrifuged after cells were gently blown. Following the removal of supernatant, the cells were rinsed and resuspended by adding a quantity of PBS solution. According to instructions of the use of the Annexin V-PE/7-ADD apoptosis kit, the cells of each group were loaded after treatment and detected by the CytoFLEX flow cytometer. Experimental data were generated by CytExpert software.

### 2.9. Detection of Cell Invasion and Migration by Transwell Assay

The experimental groups were identical to flow cytometry. Detection of invasive ability of cells was as follows: Matrigel was turned into the liquid state at 4°C overnight. Serum-free medium was mixed well with Matrigel in proper proportion, supplemented to the upper Transwell for incubation at 37°C for 4-5 h. Cell suspension was subsequently supplemented to the upper Transwell (inoculated as per 2 × 10^5^ cells/well) and medium with 10% serum to the lower Transwell. Olaparib was applied to treat in an incubator at 37°C for 48 h; then, the culture medium was rinsed using PBS. The superficial cells were wiped off with cotton balls and followed by 4% paraformaldehyde fixation. Finally, 0.1% crystal violet was applied for staining for 5-10 min. After PBS rinsing, visualization was performed via a microscope and photographed randomly in three visual fields. The invasive cells were calculated and analyzed by ImageJ software. Detection of migration ability of cells was as follows: only the upper Transwell was not covered with Matrigel; the remaining procedures were identical with the invasion experiment.

### 2.10. Statistical Methods

SPSS 22.0 software was employed for data statistics, and the data were expressed as $\overset{¯}{x}\pm \text{s}$. Analysis between both groups was subjected to Student's *t*-test, and multiple group analysis was subjected to one-way ANOVA. Variance analysis of cell proliferation and toxicity curve were adopted with repeated multiple measurements. *P* < 0.05 was considered as statistically significant.

## 3. Results

### 3.1. The Expression Level of MUS81 in BPH, PCa, and CRPC Tissues

**Figure 1:**
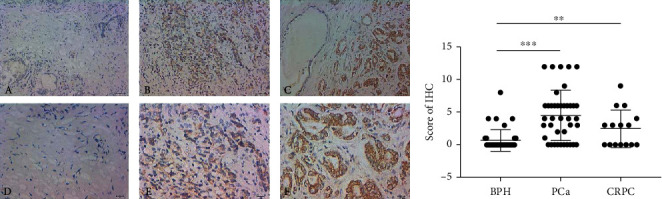
Immunohistochemical staining of MUS81 in BPH, PCa, and CRPC tissues. (a) IHC analysis of MUS81 in BPH (A and D), PCa (B and E), and CRPC (C and F) tissues. (b) Scores of IHC and analysis of MUS81 expression in BPH, PCa, and CRPC tissues. ^∗∗^*P* < 0.01 and ^∗∗∗^*P* < 0.001, versus the expression of MUS81 in BPH.

Immunohistochemical staining results indicated that positive expression of MUS81 was greatly different in BPH, PCa, and CRPC tissues, and the expression was elevated in PCa and CRPC tissues. The differences were statistically significant (Figures 1(a) and 1(b)).

### 3.2. High Expression of MUS81 in CRPC Cells

**Figure 2:**
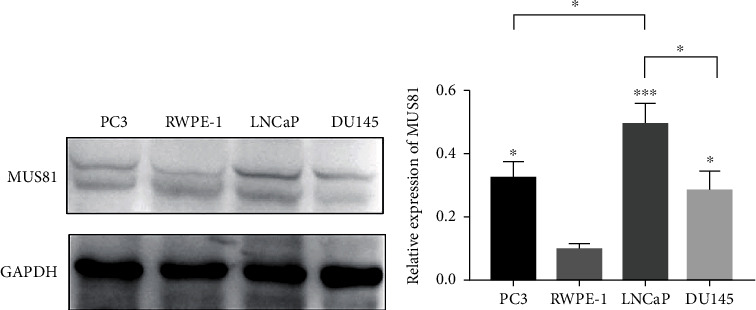
Differences in protein expression levels of MUS81 in PC3, RWPE-1, LNCaP, and DU145 cells. (a) The relative MUS81 expression in PC3, RWPE-1, LNCaP, and DU145 cells was detected via western blot. (b) MUS81 protein expression in PC3, RWPE-1, LNCaP, and DU145 cells was detected via western blot. ^∗^*P* < 0.05 and ^∗∗∗^*P* < 0.001, versus the expression of MUS81 in RWPE-1 cells.

MUS81 expressions in cancer cell LNCaP, PC3, and DU145 were higher than those in healthy human prostate epithelial cell line RWPE-1, and its expression in LNCaP cells was higher than that in PC3 and DU145 cells (Figures 2(a) and 2(b)).

### 3.3. Detection of Lentivirus Transfection Efficiency in CRPC Cells by Western Blot and PCR

**Figure 3:**
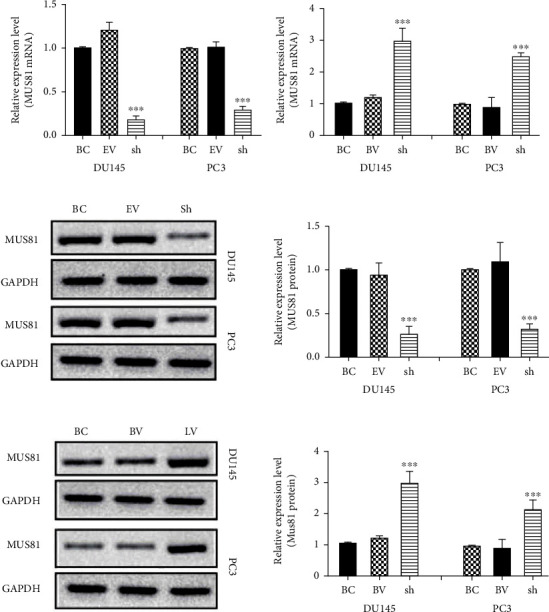
After lentivirus transfection, the expression of MUS81 in DU145 and PC3 cells. (a) The relative levels of the MUS81 mRNA transcripts after silencing MUS81. (b) The relative levels of MUS81 protein after silencing MUS81. (c) The relative levels of the MUS81 mRNA transcripts after overexpressing MUS81. (d) The relative levels of MUS81 protein after overexpressing MUS81. ^∗∗∗^*P* < 0.001 versus the no-load virus group (EV) cells and negative control group (BV) cells.

CRPC cells PC3 and DU145 were transfected with lentivirus, and four groups of treated cells were obtained. They were the MUS81 overexpressed group (LV), negative control group (BV), MUS81 silencing group (sh), and no-load virus group (EV). Results of the qRT-PCR assay demonstrated that the mRNA expression of MUS81 protein in the sh group was remarkably reduced compared with that in the EV group (Figure 3(a)), while the expression in the LV group elevated greatly compared with that in the BV group (Figure 3(c)). The results of western blot were consistent with those of qRT-PCR (Figures 3(b) and 3(d)). The previous results indicated that MUS81 overexpressed and silenced stable cell lines were successfully obtained by lentivirus transfection.

### 3.4. Effects of Overexpression and Silencing of MUS81 on CRPC Cell Proliferation and ATM Expression

**Figure 4:**
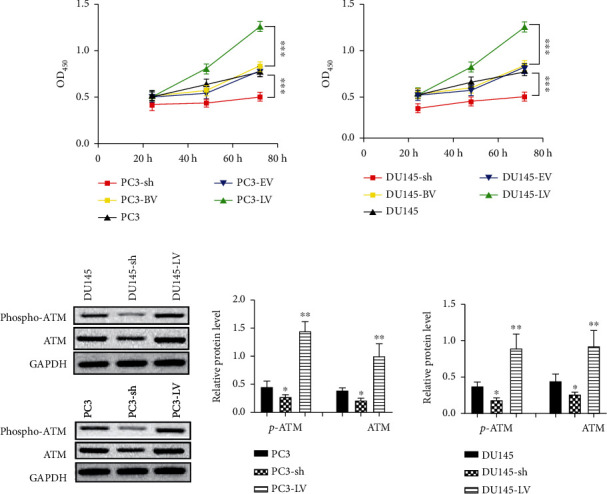
Effect of overexpression and MUS81 silencing on CRPC cell proliferation and its relationship with ATM protein levels. (a) CRPC cell proliferation. (b) The relative phospho-ATM and ATM protein expression. ^∗^*P* < 0.05 and ^∗∗^*P* < 0.01, compared with PC3 cells. ^∗∗∗^*P* < 0.001.

Cell growth was nearly unaffected for both the negative control group and the no-load virus group. The cell proliferation ability of the overexpression group was largely elevated versus the negative control group, while greatly minimized in the silence group versus the no-load virus group (Figure 4(a)). To explore the potential matrix of MUS81 action on CRPC cell proliferation, we identified ATM and p-ATM protein levels in transfected cells of each group. Results demonstrated that MUS81 silencing downregulated ATM and p-ATM protein levels, while elevating the expressions in the overexpression group (Figure 4(b)). Consequently, it was suggested that MUS81 affected the proliferation of CRPC cells, and as MUS81 expression in CRPC cells increased markedly versus healthy cells, the impact of MUS81 silencing on cells was given much priority in subsequent experiments.

### 3.5. MUS81 Silencing Promotes the Sensitivity of CRPC Cells to Olaparib

**Figure 5:**
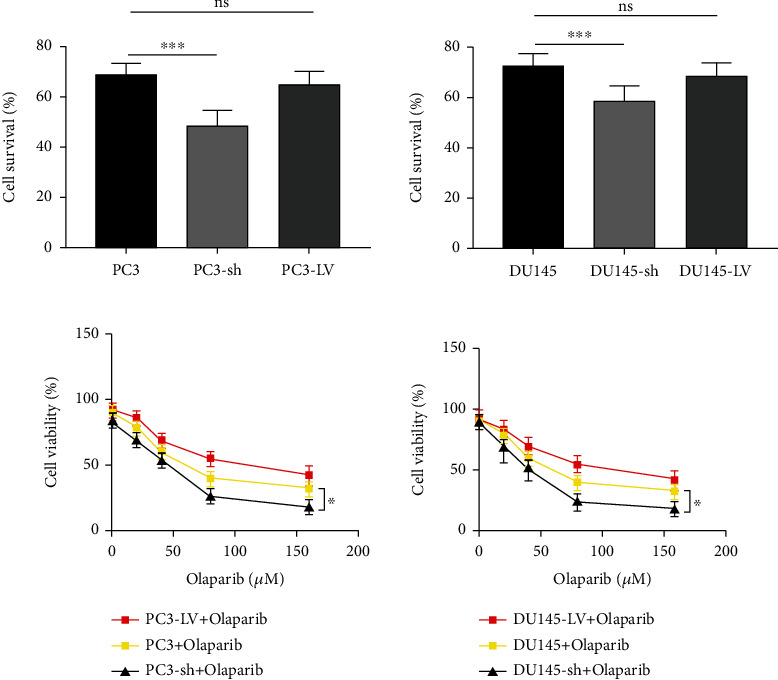
Relationship between the MUS81 expression and the sensitivity to Olaparib. (a) CRPC cell viability after 80 *μ*M Olaparib treatment. (b) Cell viability of PC3, PC3-LV, PC3-sh, DU145, DU145-LV, and DU145-sh cells at 3 × 10^3^ cells/well following Olaparib treatment at several concentrations for 48 h by CCK-8 assay. ^∗^*P* < 0.05 and ^∗∗∗^*P* < 0.001.

Cytotoxicity induced by Olaparib was subjected to the CCK-8 assay. Findings demonstrated that following 80 *μ*M Olaparib treatment for 48 h, the cell survival rate of MUS81 in both the overexpression and silence groups was decreased versus the blank control, with the silence group the most significant (Figure 5(a)). Then, we treated each group with Olaparib at different concentrations, indicating a sharp decline of cell survival rate in the MUS81 silence group. However, the survival rate of cells in the MUS81 overexpression group did not present a significant decrease versus the blank control (Figure 5(b)) indicating that MUS81 silencing enhanced the sensitivity of CRPC cells to Olaparib, the chemotherapeutic drug.

### 3.6. MUS81 Silencing Combined with Olaparib Promotes CRPC Cell Apoptosis

**Figure 6:**
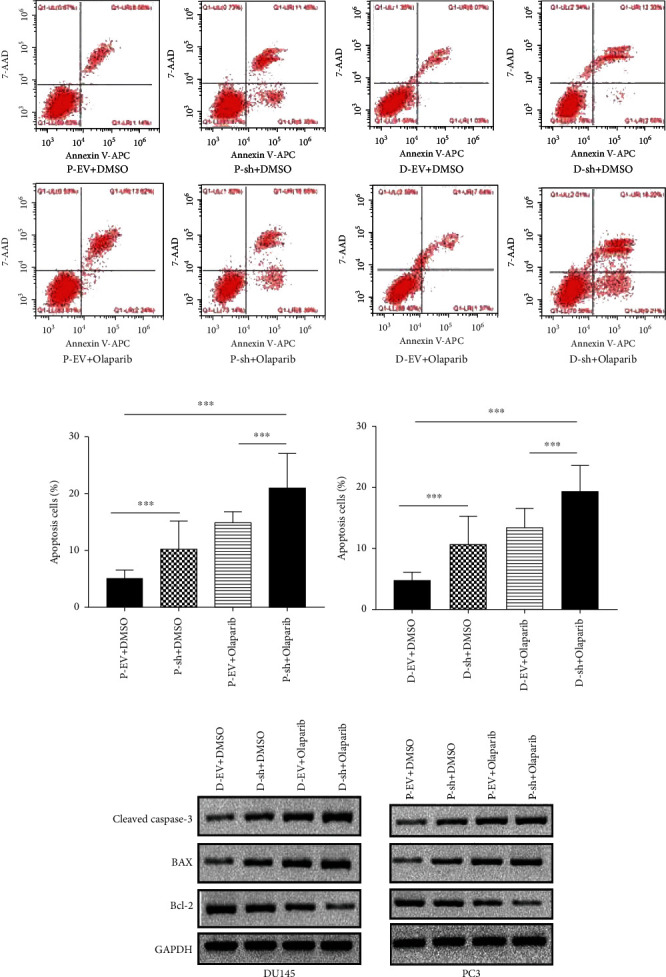
Effects of MUS81 silencing combined with Olaparib on apoptosis of CRPC cells. (a) Cell apoptosis of D: DU145, P: PC3.D-EV, D-sh, P-EV, and P-sh following 48 hours of either Olaparib or DMSO treatment by Annexin V-APC/7-AAD. (b) Apoptosis. (c) Expression of cleaved caspase-3, Bax, and Bcl-2 proteins in the CRPC cells by western blot. ^∗∗∗^*P* < 0.001.

Cells of the MUS81 silent group and no-load virus group followed either Olaparib or DMSO treatment for 48 h, results of flow cytometry indicated that the apoptosis rate of the MUS81 silent group increased markedly versus that of the no-load virus group (Figure 6(a)), and apoptosis ranked the top in MUS81 silencing combined with Olaparib (Figure 6(b)). Moreover, we also detected expressions of apoptosis-associated proteins in cells of each group by western blot. Levels of caspase-3 and BAX proteins were enhanced while that of Bcl-2 protein was significantly decreased in MUS81 silencing combined with the Olaparib treatment group versus the no-load virus group, MUS81 silenced group, and no-load virus combined with Olaparib treatment group (Figure 6(c)).

### 3.7. MUS81 Silencing Combined with Olaparib Inhibits CRPC Cell Invasion and Migration

**Figure 7:**
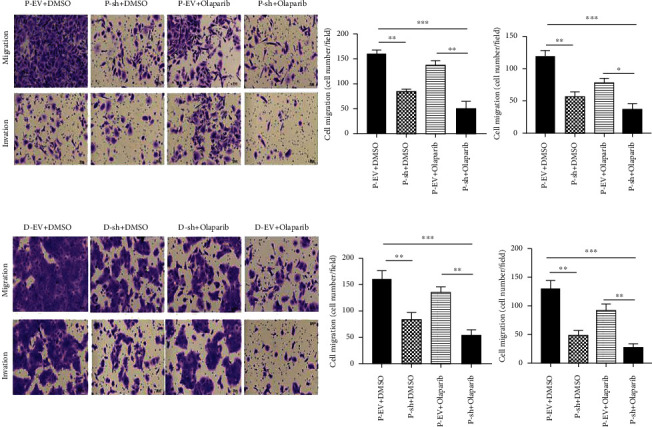
Effects of MUS81 silencing combined with Olaparib on the invasion and migration ability of CRPC cells. (a, b) The migration and invasion of D-EV, D-sh, P-EV, and P-sh following 48 hours of either Olaparib or DMSO treatment by Transwell assays. ^∗^*P* < 0.05,^∗∗^*P* < 0.01, and^∗∗∗^*P* < 0.001, versus EV-treated group.

Transwell results demonstrated that the number of cells in MUS81 silencing combined with the Olaparib group going through matrix gel and migration was the lowest (Figures 7(a) and 7(b)), indicating that MUS81 silencing combined with Olaparib could effectively inhibit CRPC cell invasion and migration.

## 4. Discussion

As a powerful DNA repair gene [8] and a member of the XPF endonuclease superfamily, MUS81 featured both high conservation and specificity. Its encoding protein was characterized by two helix-hairpin-helix motif (HhH) structures that could bind to DNA molecules [7]. Once DNA was damaged, the MUS81 protein dealt with a stagnation of replication, replication failure, and free 3′end [27, 28], so MUS81 was of great significance for DNA repair and chromosome stability. MUS81 silencing gene made mice susceptible to spontaneous tumors [12], and the expression of MUS81 decreased in liver cancer [13], colorectal cancer [15], and gastric cancer [14] as well as high malignant astrocytoma [29], which linked to tumor stage, grade, metastasis, and prognosis. In recent years, some scholars have proven that the MUS81 gene expression was remarkably upregulated in ovarian cancer [18, 19, 30], which aroused our research interest. To understand the expression of MUS81 in CRPC, immunohistochemistry and western blot assays were carried out which indicated the upregulation of MUS81 expression both in PCa and CRPC. For further exploration of the biological functions of MUS81 in CRPC, we constructed MUS81 overexpression and silent cell lines by transfecting PC3 and DU145 cells with lentivirus. The proliferation of CRPC cells was promoted following overexpression of MUS81 while inhibited after MUS81 silence. Meanwhile, apoptosis of CRPC cells was enhanced whereas the invasion and metastasis were remarkably inhibited following MUS81, indicating that the expression of MUS81 largely affected the CRPC growth. In cells with telomere function abnormality, ATM functioned as one of the activated principal DNA damage repair (DDR) signaling pathways [31, 32]. When activated ATM was phosphorylated (p-ATM), it enhanced the growth of tumor cells. Conversely, it elicited the accumulation of DNA damage and cell apoptosis. Interestingly, previous research attested that the ATM protein level in prostate cancer cells was elevated compared to heathy samples [33], and activation of ATM in prostate intraepithelial neoplasia (PIN) was recognized as a precursor of prostate cancer [34]. MUS81 silencing inactivated ATM/Chk2, thereby enhancing the sensitivity of breast cancer cell MCF-7 to cisplatin [17]. We studied the relationship between the expressions of MUS81, ATM, and p-ATM in transfected cells. Findings indicated that overexpression of MUS81 upregulated the expression of ATM/p-ATM and MUS81 silence downregulated the expression, thereby suggesting that MUS81 silencing inhibited the growth of CRPC cells by downregulating the expression of ATM/p-ATM, but the specific mechanism still needs further investigations.

As a PARP inhibitor, Olaparib caused DNA repair disorder and damage accumulation, resulting in tumor cell apoptosis. Findings in ovarian cancer revealed that MUS81 silencing enhanced the sensitivity of cancer cells to Olaparib [18]. A recent phase III clinical study exhibited that Olaparib prolonged progression-free survival and improved objective remission rate and pain duration in CRPC patients [25]. Some literature [35, 36] stated that tumor cells with ATM deficiency were more sensitive to Olaparib, which prevented DNA double strands from repairing and elicited damage accumulation, leading to cell apoptosis ultimately. We studied the role of MUS81 combined with Olaparib in CRPC cells. Results indicated that MUS81 silencing combined with Olaparib significantly inhibited cell proliferation, accelerated cell apoptosis, mediated cell invasion, and migration, and MUS81 silencing could enhance the CRPC cell sensitivity to Olaparib. To sum up, our results revealed that MUS81 could influence CRPC growth by altering levels of ATM, and MUS81 silencing could promote the cell sensitivity to Olaparib, suggesting that MUS81 could be used as an index to predict drug resistance of tumor patients to Olaparib, which was expected to become a new gene target for CRPC therapeutic options.

## 5. Conclusions

The expression of MUS81 could be upregulated in CRPC, which mediated the growth of tumor cells through ATM. MUS81 silencing enhanced the CRPC cell sensitivity to Olaparib, and the combination remarkably inhibited cell proliferation, invasion, and migration thereby accelerating cell apoptosis. This research provides evidence for predicting drug resistance of tumor patients to Olaparib by MUS81, enabling MUS81 to become a novel gene target for the treatment of CRPC.

## Data Availability

The data used to support the findings of this study are included within the article.

## Additional Points

*Highlights*. (1) The expression of MUS81 indicated marked upregulation in PCa and CRPC tissues. (2) MUS81 silencing enhanced the CRPC cell sensitivity to Olaparib. (3) MUS81 become a novel gene target for the treatment of CRPC.
